# Detrimental Effects of Anti-Nucleocapsid Antibodies in SARS-CoV-2 Infection, Reinfection, and the Post-Acute Sequelae of COVID-19

**DOI:** 10.3390/pathogens13121109

**Published:** 2024-12-15

**Authors:** Emi E. Nakayama, Tatsuo Shioda

**Affiliations:** Department of Viral Infections, Research Institute for Microbial Diseases, Osaka University, Suita 565-0871, Japan; emien@biken.osaka-u.ac.jp

**Keywords:** antibody-dependent enhancement, nucleocapsid, COVID-19, post-acute COVID-19 syndrome, long-COVID, broadly neutralizing antibody

## Abstract

Antibody-dependent enhancement (ADE) is a phenomenon in which antibodies enhance subsequent viral infections rather than preventing them. Sub-optimal levels of neutralizing antibodies in individuals infected with dengue virus are known to be associated with severe disease upon reinfection with a different dengue virus serotype. For Severe Acute Respiratory Syndrome Coronavirus type-2 infection, three types of ADE have been proposed: (1) Fc receptor-dependent ADE of infection in cells expressing Fc receptors, such as macrophages by anti-spike antibodies, (2) Fc receptor-independent ADE of infection in epithelial cells by anti-spike antibodies, and (3) Fc receptor-dependent ADE of cytokine production in cells expressing Fc receptors, such as macrophages by anti-nucleocapsid antibodies. This review focuses on the Fc receptor-dependent ADE of cytokine production induced by anti-nucleocapsid antibodies, examining its potential role in severe COVID-19 during reinfection and its contribution to the post-acute sequelae of COVID-19, i.e., prolonged symptoms lasting at least three months after the acute phase of the disease. We also discuss the protective effects of recently identified anti-spike antibodies that neutralize Omicron variants.

## 1. Introduction

**Figure 1:**
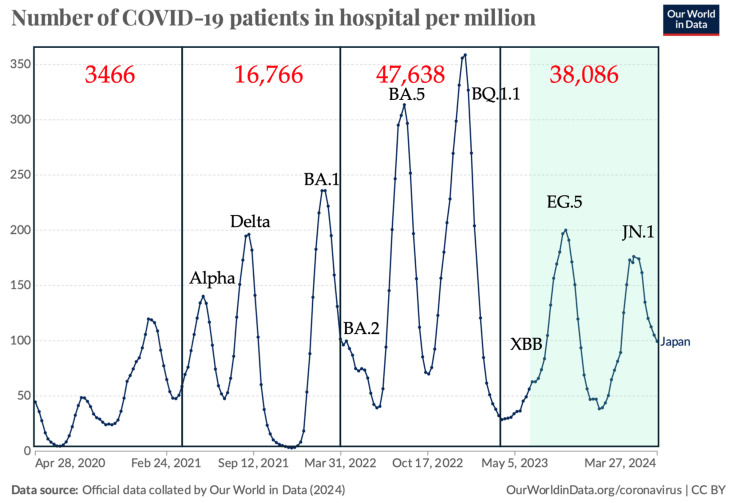
Number of hospitalized patients and COVID-19-related deaths in Japan (April 2020–March 2024). Data were modified from official statistics collated by Our World in Data, “COVID-19, hospitalizations” [44]. The numbers in red represent the official death counts sourced from the Japanese Ministry of Health, Labour and Welfare [45] for each fiscal year from April 2020 to the end of March 2024. The Japanese government changed its COVID-19 case counting policy from including all to only limited cases reported from selected hospitals on May 8, 2023. During the period highlighted in pale green, it is speculated that the actual number of hospitalized patients may be higher than that depicted in this graph. The major variants reported during each surge, as identified by the National Institute of Infectious Disease in Japan, are also noted [46].

**Figure 2:**
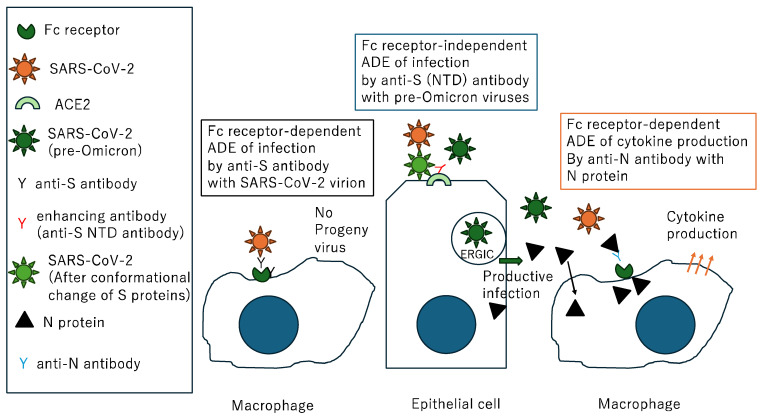
The schematic mechanisms of antibody dependent enhancement (ADE) of SARS-CoV-2. (**Left**) The Fc receptor-dependent ADE of infection is well documented in dengue virus infection. In the case of SARS-CoV-2 infection, it was observed only in the artificial cells ectopically expressing Fc receptors or ACE2/TMPRSS2. (**Middle**) The Fc receptor-independent ADE of infection is caused by a conformational change in S proteins upon antibody binding. Progeny virions bud into ER-Golgi intermediate compartment (ERGIC) and most N proteins are located in the cytoplasm of infected cells and released by cell death. (**Right**) ADE of cytokine production is caused by translocation of the N protein and anti-N antibody complex via Fc receptors on the surface of macrophages. The blue circles denote nuclei of cells.

The Severe Acute Respiratory Syndrome Coronavirus 2 (SARS-CoV-2) pandemic was first reported in Wuhan, China, at the end of 2019 [1]. The disease, named Coronavirus Disease 2019,COVID-19, was primarily characterized by Acute Respiratory Distress Syndrome (ARDS) [2]. Two doses of a vaccine based on the Wuhan strain were demonstrated to be highly effective against infection, hospitalization, and death due to the Delta variant in 2021 [3]. Despite the global surge of the less virulent Omicron variants (PANGO lineage B.1.1.529 subvariant BA.1), so-called “hybrid immunity”, i.e., immunity developed through a combination of SARS-CoV-2 infection and vaccination, was believed to protect individuals from further infection [4]. Symptoms associated with reinfection in 2022 were generally not significantly different to those of the initial infection or were relatively mild [5,6]. However, multiple surges of new Omicron subvariants such as BA.2 [7], BA.5 [8], BQ.1.1 [9], XBB1.5 [10], EG.5 [11], and JN.1 [12] continued to cause deaths by pneumonia or ARDS [13], heart failure [14], brain infarction [15], and severe exhaustion, especially among the unvaccinated elderly population. For example, more individuals died from Omicron variants in 2022 than from Delta in 2021, as shown in Figure 1. Each subsequent SARS-CoV-2 infection increases the risk of developing chronic health issues like diabetes [16,17], kidney disease [18], organ failure, and even mental health problems [19,20,21]. Following the introduction of antiviral drugs targeting RNA polymerase [22,23,24] and protease [25,26] for acute SARS-CoV-2 infections, post-acute sequelae of COVID-19 (PASC) remains a significant concern, diagnosed by symptoms such as brain fog [27] and fatigue [28] that last at least 3 months after infection [29,30]. WHO defined PASC as the continuation or development of new symptoms three months after the initial SARS-CoV-2 infection, with over 200 different symptoms lasting for at least 2 months with no other explanation [31]. This means there are no specific laboratory markers, and symptoms were based on patient complaints. Multiple studies now suggest that even seemingly mild cases of COVID-19 can lead to an increased risk of PASC among relatively young populations [32,33,34]. Why is this occurring? One hypothesis is that the antibody against the spike (S) protein, provided by vaccination or infection, might trigger antibody-dependent enhancement (ADE) of infection [35]. However, this is unlikely to be the case, as ADE of infection typically occurs when the virus replicates in cells that express Fc-receptors, such as macrophages, which capture the antibody–virus complex. This mechanism is observed in the dengue virus and in only artificial situations of SARS-CoV-2, in which Fc receptors were transduced into permissive BHK cells or ACE2 and TMPRSS2 were transduced into Fc receptor-expressing myeloid cells, as previously discussed [36] (Figure 2). Furthermore, anti-S antibodies that induce conformational changes in the S protein and enhance infection of the original strain [37,38] are unable to bind to the amino-terminal domain (NTD) of the S protein in the Omicron variant [39]. Furthermore, it was demonstrated that both the antibodies causing Fc receptor-dependent ADE and those causing the conformational change-induced ADE failed to enhance virus growth in antibody-infused and subsequently SARS-CoV-2-challenged rhesus monkeys [37,38]. The S protein in SARS-CoV-2 virions is proposed to enhance inflammation effects, leading to local platelet stimulation and subsequent activation of the coagulation cascade [40], as well as binding to fibrin [41] and promoting inflammation via Toll-like receptor 2 [42]. Apart from myocarditis [43], there is no clear evidence that the S protein itself acts as a toxin or plays a direct agonist/antagonist role. In this review, we discuss another structural protein, the nucleocapsid (N), and the anti-N antibodies. We also explore recent progress in the protective effects of anti-S antibodies.

## 2. Detrimental Effect of N Protein of SARS-CoV-2

The N protein of SARS-CoV-2 has functional RNA-binding domains at both its amino- and carboxyl-termini, which encapsulate the viral genome within the virion [47,48]. As reviewed by Yu et al. [49], the N protein modulates the innate immune response in infected cells and induces inflammation. Expression of the N protein, but not the S protein, leads to a marked increase in the production of proinflammatory cytokines, such as interleukin (IL)-6, IL-12, IL-1β, and tumor necrosis factor (TNF)-α [50]. Zhang et al. [51] previously reported that the expression of the carboxyl terminal domain (CTD) of the N protein alone is sufficient for inducing IL-6. In contrast, the S protein, anchored to the endoplasmic reticulum membrane via its CTD, remains less mobile [52]. Soluble N protein, packed in the cytoplasm of infected cells, is released easily upon cell death. This released N protein binds to mannan-binding lectin-associated serine protease 2, causing complement hyperactivation via the lectin pathway in the bloodstream [53]. On the other hand, activation of the NOD-, LRR-, and pyrin domain-containing protein 3 inflammasome by intracellular N protein has been reported to lead to cytokine storms [54]. According to Teuwen et al. [55], both complement activation and hyperinflammation lead to blood vessel damage and multiple organ failure. In addition, cerebrospinal fluid (CSF) samples from patients with neural symptoms during acute infection were typically negative for SARS-CoV-2 RNA, yet the N protein was detected in the CSF in 89% of 35 patients, compared to only 3% for the S protein [56]. This suggests that the soluble N protein has a high potential to cross the blood–brain barrier and reach the central nervous system, since elevated IL-8 would loosen the gap between vascular endothelial cells. Furthermore, IL-6, IL-8, and TNF-α are elevated in severe cases [57,58,59,60,61,62,63], and TNF-α in particular can trigger senescence-like cell-cycle arrest in neighboring uninfected cells in a paracrine manner [64].

## 3. Adverse Effects of Anti-N Protein Antibody

The systemic cytokine profiles observed in patients with severe COVID-19 bear similarities to those seen in cytokine release syndromes, such as macrophage activation syndrome [65]. In monocytes, the uptake of SARS-CoV-2 by phagocytosis has been confirmed by staining for N proteins, but they do not progress to productive infection due to pyroptosis [66]. We previously reported that macrophages derived from peripheral blood monocytes cannot produce progeny viruses [67], a finding consistent with other reports [68,69]. Similarly, abortive infections of macrophages have been documented with SARS-CoV-1 [70,71] and Middle East respiratory syndrome (MERS) -CoV [72]. In contrast, the addition of N protein can induce massive cytokine production, including IL-6, IL-8 and TNF-α from macrophages [67]. The addition of a comparative amount of purified soluble S protein does not sufficiently induce IL-6 production. However, the addition of anti-N mouse monoclonal antibody subclass IgG1, which can bind to Fcγ-receptors of humans, but not mouse IgG2b, enhances IL-6 production from the myeloid cell line K-ML2 [73] in the presence of N protein. This indicates Fc receptor-dependent ADE of cytokine production by N protein. Patient sera from severe cases induced more IL-6 compared to that from mild cases [67]. However, our study in Japan has one critical problem with the date of sampling. Briefly, patients with severe cases were typically transferred to tertiary hospitals for intensive care for approximately 8 to 10 days after symptom onset, while samples from mild cases were collected at the time of diagnosis—approximately 3 to 4 days after symptom onset in Japan. To normalize the timing of serum sampling, we collaborated with a private hospital in Bangladesh, which was frequented by wealthier patients for primary care. This facility, equipped with an intensive care unit, also functions as a tertiary care hospital. Consequently, we managed to collect patient serum approximately 3 days after symptom onset during an Omicron surge. We found that the serum from patients who subsequently developed severe pneumonia in their disease course induced more IL-6 compared to the serum from patients who recovered quickly, particularly when we added K-ML2 cells in the presence of N protein [74]. The concentration of anti-N antibodies in patient serum, measured using an in-house ELISA that employed full-length N protein, correlated with the ability of serum to induce IL-6 and IL-8 from K-ML2 cells in the presence of N protein [74].

Although typically considered to be localized within the cytosol, N proteins have been detected on the surfaces of live cells [75]. Released by SARS-CoV-2–infected cells, N protein binds to neighboring cells through electrostatic affinity to heparan sulfate and heparin [75]. Furthermore, anti-N antibodies bound to the surface of cells expressing N protein can activate cells that express Fc receptors. This activation strongly enhances antibody-dependent natural killer cell activity via non-spike antibodies, such as those targeting the N protein [76]. This suggests that pre-existing anti-N antibodies may promote bystander cell death through antibody-dependent cellular cytotoxicity.

ADE phenomena that enhance disease severity have been documented during vaccine development for respiratory syncytial virus, SARS-CoV-1, and MERS-CoV as vaccine-associated enhanced diseases [77]. It has been reported that immunization with the S protein of the SARS-CoV-1 can protect mice against challenge virus infection, whereas mice immunized with the N protein develop more severe pneumonia compared to unimmunized mice following challenge virus infection [78]. Elevated serum levels of IL-4 and IL-5 in mice immunized with the N protein suggest that a type-1 helper T-cell (Th1) response was important for vaccine safety against pulmonary diseases. Additionally, increased levels of IL-6 were also observed in mice immunized with the N protein of the SARS-CoV-1 [78].

In a study of SARS-CoV-2-infected individuals in an intensive care unit (ICU), Sun et al. [79] measured N and S protein-specific IgMs and IgGs. They found that the IgG response to the S protein was significantly higher in non-ICU COVID-19 patients than in ICU patients in the third week after patient recovery. In contrast, the IgG response to the N protein was significantly higher in ICU patients than in non-ICU patients from the first week, coinciding with the development of severe pneumonia [79]. The unusual early increase in IgG levels immediately after infection led to speculation that this may be due to cross-reactions of antibodies from common cold coronaviruses recognizing the SARS-CoV-2 N protein. A longitudinal comparison between COVID-19 patients with and without pneumonia revealed that anti-N antibody levels in pneumonia patients were higher than those in non-pneumonia patients even after one year after the onset of symptoms [80]. Given the similarities in the N protein among common cold coronaviruses [81], refined techniques are necessary to measure anti-SARS-CoV-2 N-specific antibodies and avoid false positives in diagnosis. Furthermore, these cross-reacting antibodies can induce ADE of cytokine production [79], which explains why the elderly population, more frequently exposed to common cold coronaviruses, experienced severe illness at the onset of the pandemic [82].

## 4. Risks of Reinfection and PASC, Commonly Referred to as ‘Long-COVID’

Except for dengue virus infection, in which the ADE of infection may be involved in the pathogenesis of severe dengue upon the secondary infection [83], it is generally believed that reinfection results in a milder disease course than the primary infection, primarily due to the immunity developed during the previous infection. However, data from 2022, including weekly counts of SARS-CoV-2 reinfections, total infections, and COVID-19-associated hospitalizations and deaths reported by 18 US jurisdictions, indicate that reinfections continued to cause severe diseases during repeated Omicron surges [84]. Additionally, a study using the US Department of Veterans Affairs COVID-19 database showed a higher incidence of hospitalization or death after reinfection (7.31 per 1000 person-days) compared to that following breakthrough infections (4.69 per 1000 person-days) [85]. Bowe et al. [86] examined whether SARS-CoV-2 reinfection increases health risks. Specifically, they found the increased cumulative risks of hospitalization, cardiovascular disease, coagulation and hematological abnormalities, diabetes, fatigue, gastrointestinal symptoms, kidney dysfunction, mental health disorders, musculoskeletal dysfunction, neurological disorders, and pulmonary dysfunctions associated with two, three, or more infections, compared to those in noninfected controls or after one infection. Although there were no data on anti-N antibodies in this study, it seems reasonable to assume that levels of anti-N antibodies were lower in the primary infection than after multiple infections. The heightened health risks following secondary infections could be attributed to: (1) cumulative chronic organ dysfunction from vascular infarctions, (2) exacerbated problems from hyperinflammation in the presence of anti-N antibodies, or (3) a combination of both.

With respect to the PASC, an increasing number of patients, despite resolution of many acute viral infection symptoms, continue to experience ongoing complications at least three months post-infection [87,88]. These PASC patients vary markedly in terms of both manifestation and severity, ranging from anosmia and fatigue to joint pain and dyspnea. Recent findings from the RECOVER adult cohort study indicated that the estimated proportion of PASC positivity was higher among participants who were reinfected during the Omicron phase compared with those reporting a single infection (20% vs. 9.7%, respectively), whereas rates were similar in the pre-Omicron phase (33% vs. 37%, respectively) [89]. Earlier reports from the UK COVID-19 Infection Survey indicated a relatively low risk of reinfection [90]. In contrast, The Canadian COVID-19 Antibody and Health Survey showed that Canadians reporting two known or suspected COVID-19 infections were 1.7 times more likely to report PASC than those with only one known or suspected infection (25.4% vs. 14.6%), and the likelihood increased to 2.6 times for those with three or more infections (37.9% vs. 14.6%) [91]. Furthermore, analyses of two large primary care electronic health records datasets from England, UK, and Catalonia, Spain, confirmed that persistent symptoms were more common after reinfection than after a first infection [92]. All post-acute COVID-19 symptoms were reported more frequently following reinfection than after the first infection, even when matched by age, sex, and date of infection [92]. Similar trends have been reported in Quebec, Canada [93], and China [94], and are expected to be reported in the United States [95].

Among a subset of patients with PASC who presented with shortness of breath, cough, and hypoxemia, detectable abnormalities were observed on computed tomography imaging of the lung [96]. Fourteen biomarkers were found to be increased in bronchoalveolar lavage fluid, including monocyte and T cell chemokines (CCL2, CCL7, CCL4 and CCL15), inflammatory cytokines (IL-1α, IL1-receptor antagonist, IL-6, and IL-8), CXCL13 (involved in B lymphocyte recruitment), epidermal growth factor (promotes epithelial repair), CXCL5 (neutrophil recruitment during repair), CCL11 (eosinophil recruitment), soluble CD40 ligand (sCD40L; affects various immune cell activations), and the apoptosis ligand TRAIL [96]. These findings suggest that monocyte-derived macrophages are recruited to the alveolus in proportion to the severity of injury. Their findings also showed that cytokines released from macrophages to recruit neutrophils are related to disease severity [97]. Furthermore, Grant et al. [98] reported that COVID-19 patients experience a higher cumulative systemic exposure to lung-derived inflammatory cytokines during their illness compared to patients with other infections, such as bacterial infections, suggesting that a prolonged cytokine storm may drive more severe post-acute sequelae in COVID-19 survivors.

It was also reported that PASC patients exhibiting cardiovascular symptoms, such as chest pain and heart palpitations, showed significantly increased levels of IL-12, IL-1β, MCP-1, and IL-6 in their plasma compared to those that had recovered [99]. Additionally, plasma and immune cell profiling have facilitated the stratification of PASC into inflammatory and non-inflammatory types [100]. Inflammatory PASC, identifiable through a set of 12 blood markers including IL-8, shows evidence of ongoing neutrophil activity. In particular, anti-N antibodies were enriched in the inflammatory PASC cohort, with significant increases in both IgA and IgG responses observed more than 120 days post-diagnosis [100]. It remains unclear whether these elevated levels of anti-N antibodies result from a persistent infection with SARS-CoV-2 or from pre-existing anti-N antibodies at the time of infection.

## 5. Concerns Regarding the Use of N Protein as a Vaccine Antigen

In the context of COVID-19, there are two main types of vaccine against SARS-CoV-2: (1) those targeting only the S protein, including mRNA-based vaccines [101,102,103], adenovirus vector vaccines [104], and recombinant protein vaccines [105]; and (2) those targeting the whole virion, which includes the N protein. Given the propensity of RNA viruses to rapidly evolve escape variants, there is considerable debate over whether proteins other than the S protein should be used as vaccine antigens. Inactivated, whole-virion vaccines have been shown to induce lower neutralization titers compared to other platforms [106,107,108]. It is documented that K986P/V987P mutations introduced in the S protein are important for stably exposing the receptor-binding domain, resulting in a higher production of neutralizing antibodies [109]. These mutations have been incorporated into mRNA-based and recombinant protein vaccines, but not into adenovirus vector or whole inactivated virion vaccines. Inactivated whole-virion vaccine makers in China and India did not prepare vaccines for Omicron variants probably due to the lower efficacy than mRNA vaccines [110]. During the Omicron surge, detrimental effects on protection against self-reported symptoms were observed more with inactivated whole-virion vaccination, compared to at least one non-inactivated vaccine (OR = 1.942, 95% CI: 1.093–3.448 vs. OR = 0.428, 95% CI: 0.226–0.812, respectively) [111]. This suggests that anti-N antibodies may enhance the disease when levels of anti-S antibodies are insufficient for neutralizing Omicron variants, although involvement of antibodies against another viral antigen could not be excluded for the enhancement of symptoms. Comparing the frequency of PASC across studies is challenging, but the reported frequency of PASC in China, where whole inactivated vaccines of SARS-CoV-2 were commonly used until 2022 [112,113], is relatively high, with 57.3% (2754 of 4804) of patients experiencing symptoms such as fatigue, cough, and muscle pain [114].

To enhance T cell immunity across epitopes less frequently altered in SARS-CoV-2 variants, the vaccine developer BioNTech (Mainz, Germany) designed BNT162b4, an mRNA vaccine component that is intended to complement BNT162b2, the S protein-encoding vaccine [115]. BNT162b4 encodes variant-conserved, immunogenic segments of the SARS-CoV-2 N, membrane protein, and ORF1ab proteins. This strategy specifically selects segments enriched in both reported and predicted CD8^+^ and CD4^+^ T-cell epitopes. For the N protein, only the C-terminal half is included [115]. BNT162b4 is expected to demonstrate better efficacy than the whole inactivated vaccine described above, since BNT162b4 was designed to elicit T cell immunity, which is less efficiently induced by whole inactivated vaccines. Although authors measured anti-S but not anti-N antibodies, concerns remain that BNT162b4 might also induce detrimental anti-N antibodies, given that antibodies targeting the C-terminal half of the N protein have been shown to enhance IL-6 production induced by the N protein [36].

## 6. Recent Advances in Anti-S Antibody Research

As the final chapter, we discuss the protective and possible detrimental effects of antibodies against another major viral component, spike protein.

**Figure 3:**
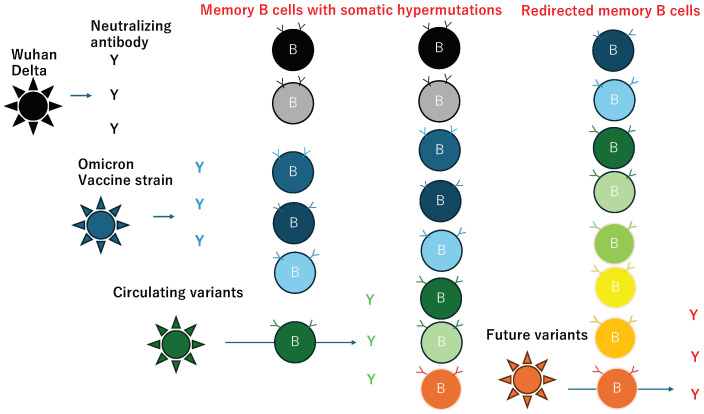
The fight against mutated viruses by antibody and memory B cell repertories. Different colors show the different antibody and B cell clones. “Y” s and Y on the B cells denote the antibodies in serum and B cell receptors on the surface of B cells, respectively. The short arrows represent the protective effect of antibodies in plasma, which can neutralize the viruses previously infected or vaccinated strains. The long arrows represent the process of stimulation of the selected B cell maturation followed by efficient neutralization of the variants by the produced antibodies.

The rapid evolution of SARS-CoV-2 has led to the emergence of neutralizing antibody escape variants, causing a continual shift in the dominant strain and frequent reinfections. The strains used in booster vaccines typically lag behind the viruses currently in circulation. However, the B-cell memory repertoire is equipped to prepare for potential future mutants through somatic hypermutation in the immunoglobulin gene [116], which is known to produce so-called ‘broadly neutralizing antibodies’ (bnAb). These bnAbs can neutralize not only the wild type, but also various mutants [117]. This is distinct from antibodies that react with conserved epitopes located in the S2 region [118]. The bnAbs can cover the receptor binding domain of Omicron and neutralize more variants [119,120,121,122,123,124,125,126,127,128,129,130,131]. In the context of HIV infection, it typically requires almost three years to develop bnAbs [132]. However, repeated exposures to Omicron have redirected the evolution of SARS-CoV-2–specific memory B cells from the original Wuhan strain toward the latest variants [133]. Consequently, we observed a slightly elevated neutralizing activity against BA.2 in pooled plasma from blood donors collected during the third dose vaccination against the Wuhan strain before the arrival of the BA.2 surge [134]. This adaptation of B cells in preparation for upcoming variants, illustrated in Figure 3, is promising; however, it is important to note that while B cells stored with the memory phenotype can produce significant amounts of antibodies to control the disease, this process requires several days. If the exposed viral load is high, then anti-S antibodies circulating in the body may not be sufficient for preventing the spread of infection from the initially infected cells to others. An increase in infected cells results in a higher release of N protein, which can trigger the induction of anti-N antibodies alongside N antigen, potentially leading to ADE of cytokine production.

In this review, we cast doubt on the influence of the anti-S antibody-dependent enhancement of infection in infected individuals. However, there are rare cases in which ADE of infection may significantly affect the course of the disease. Regarding human genome polymorphisms, it has been reported that the rs13050728 T/T genotype located in the *IFNAR2* locus is a risk factor for severe COVID-19 [135]. This genetic variation results in an *IFNAR2*-*IL10RB* readthrough transcript, which expresses an IFNAR2-IL-10RB hybrid receptor. Typically, macrophages express low levels of angiotensin-converting enzyme 2 (ACE2) [136], the receptor for SARS-CoV-2 infection [137]. However, alveolar macrophages that express the IFNAR2-IL-10RB hybrid receptor upregulate ACE2 expression under IL-10 stimulation, becoming more susceptible to SARS-CoV-2 replicative infection [135]. Consequently, these specific IL-10-induced alveolar macrophages may potentiate anti-S antibody-dependent enhancement of infection. Since IL-10 is a consequence of inflammation, reducing exposure to the virus is crucial for preventing severe disease, as described above.

## 7. Discussion

**Figure 4:**
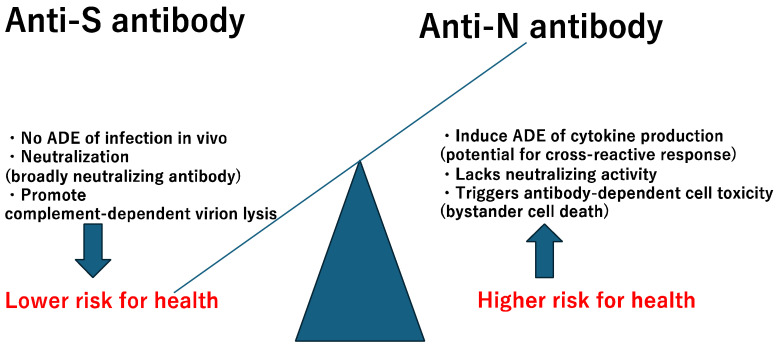
Characteristics of anti-spike (S) and anti-nucleocapsid (N) antibodies. ADE: Antibody dependent enhancement.

It is important to consider the two distinct types of antibodies in the context of infection and reinfection. The unique properties of anti-S and anti-N antibodies are illustrated in Figure 4.

It is also important to note that non-neutralizing or even infection-enhancing antibodies [37] can still deplete virions in vivo through virolysis mechanisms [138]. These mechanisms activate the complement system, creating a so-called membrane attack complex like holes on the virion surface membrane to inactivate the enveloped viruses. Although the anti-S-dependent enhancement of infection was observed in vitro [139], there is no clear evidence of ADE of infection in vivo, as the enhancing antibodies injected into rhesus monkeys also provided protection from viral challenges [37]. Therefore, booster vaccinations to increase the amount of anti-S antibody is another key factor in reducing the risk of refection. Inoue et al. [128] suggested that the occupation of the epitope by antibodies against older virus types could drive the evolution of the B-cell receptor gene, which is identical to the immunoglobulin gene to bind other strains. Further studies are required to determine whether frequent booster vaccinations, such as every 3 or 6 months, are more effective than annual boosters.

**Figure 5:**
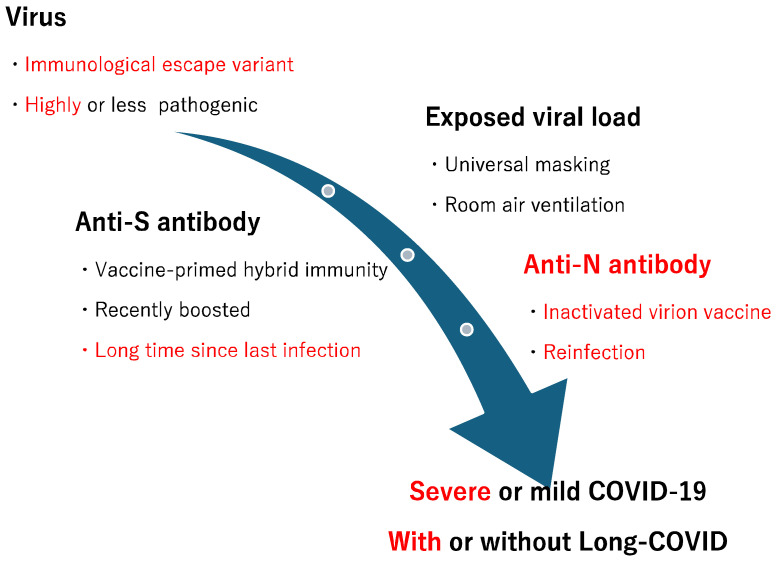
Factors influencing the severity of COVID-19. The negative factors for health are shown in red.

Lind et al. [140] discovered that hybrid immunity significantly reduced the risk of infection among residents with moderate or no documented exposures, but not among those with close exposures, during both the Delta and Omicron phases. This suggests that universal masking [141,142,143,144,145,146], ventilation of room air [147,148], and other procedures that reduce community viral load could facilitate the protective effects of hybrid immunity (Figure 5).

## 8. Conclusions

It is now evident that SARS-CoV-2 reinfection poses significant health risks, though studies specifically examining the role of anti-N antibodies are still limited. While treatments for PASC are still under development [149], preventing breakthrough infection and reinfection remains preferable.

## 9. Future Directions

In addition to the effect of cytokine storms, multiple factors are responsible for PASC [150]. These factors include prolonged infection [151], exhausted T cell responses [152], autoimmune status including the presence of autoimmune antibodies [153], reactivation of Epstein–Barr virus [154], and low levels of cortisol [154] and serotonin [155]. Further studies are necessary to elucidate the involvement of anti-N antibodies in the risk of SARS-CoV-2 reinfection and PASC. Since most of the studies were conducted in North American and European countries, more studies in Asian and African countries are needed. Additionally, comprehensive epidemiological studies are needed to assess the long-term effects of vaccination on disease outcomes and reinfection rates.
